# National Hospital for the Paralysed and Epileptic

**Published:** 1892-06-04

**Authors:** 


### NATIONAL HOSPITAL FOR THE PARALYSED AND EPILEPTIC.

The annual general meeting of the governors and subscribers was held on the 26th ult., the President of the Hospital, the Duke of Westminster, in the chair.

The annual report, which we have already noticed in our columns, having been read by the Secretary (Mr. B. Burford Rawlings),

The Chairman moved its adoption. He said the report began and ended with references to the extended requirements of the hospital. As it was, so many would-be patients had to be refused for want of adequate space, that the board very reasonably to his mind, looked to an enlargement of the premises in a certain direction, which was not to be mentioned. Of course, in so doing they would not fail to remember that if the hospital was enlarged there came the question of increased means (which were not always so readily forthcoming) to maintain the wards and to maintain the increased number of patients. That was a difficulty, and it called for the earnest consideration of the board. The amount of good which had been done by the hospital during the past year was far larger than it had ever accomplished previously, a larger number of patients having been treated at a less cost for maintenance. (Applause.)

Sir James Crichton Browne, in seconding, very earnestly recommended the hospital to the support of all those who were charitably disposed. He had had a very considerable acquaintance with the working of that hospital, and that justified him in affirming very positively that there was no hospital in that country more deserving of support, nor was there any hospital in which medical science was grappling more closely and effectively with the difficult problems of disease than the National Hospital for the Paralysed and Epileptic. (Applause.) Every medical man who was interested in the study and treatment of disorders of the nervous system, not only in England, but all over the world, had to keep an eye on that hospital, for there was assembled there a small but distinguished body of physicians and surgeons, who, acting as pioneers through the dismal forest of neurotic disorders, had already opened up vistas of hopefulness in directions where everything before was dark and dismal. (Applause.)

The retiring members of the board, the Hon. H. D. Ryder and Mr. A. L. Wheeler, and the auditors, were re-elected. Ten pensioners were elected upon the fund for incurables, and various votes of thanks were passed.

